# Serum Free Cultured Bone Marrow Mesenchymal Stem Cells as a Platform to Characterize the Effects of Specific Molecules

**DOI:** 10.1371/journal.pone.0012689

**Published:** 2010-09-10

**Authors:** Leonardo Solmesky, Sharon Lefler, Jasmine Jacob-Hirsch, Shlomo Bulvik, Gideon Rechavi, Miguel Weil

**Affiliations:** 1 Department of Cell Research and Immunology, Faculty of Life Sciences, Tel Aviv University, Tel Aviv, Israel; 2 Hematology Department, Laniado Hospital, Netanya, Israel; 3 Cancer Research Center, Sheba Medical Center, Tel Hashomer, Ramat Gan, Israel; University of Texas MD Anderson Cancer Center, United States of America

## Abstract

Human mesenchymal stem cells (hMSC) are easily isolated from the bone marrow by adherence to plastic surfaces. These cells show self-renewal capacity and multipotency. A unique feature of hMSC is their capacity to survive without serum. Under this condition hMSC neither proliferate nor differentiate but maintain their biological properties unaffected. Therefore, this should be a perfect platform to study the biological effects of defined molecules on these human stem cells. We show that hMSC treated for five days with retinoic acid (RA) in the absence of serum undergo several transcriptional changes causing an inhibition of ERK related pathways. We found that RA induces the loss of hMSC properties such as differentiation potential to either osteoblasts or adipocytes. We also found that RA inhibits cell cycle progression in the presence of proliferating signals such as epidermal growth factor (EGF) combined with basic fibroblast growth factor (bFGF). In the same manner, RA showed to cause a reduction in cell adhesion and cell migration. In contrast to these results, the addition of EGF+bFGF to serum free cultures was enough to upregulate ERK activity and induce hMSC proliferation and cell migration. Furthermore, the addition of these factors to differentiation specific media instead of serum was enough to induce either osteogenesis or adipogenesis. Altogether, our results show that hMSC's ability to survive without serum enables the identification of signaling factors and pathways that are involved in their stem cell biological characteristics without possible serum interferences.

## Introduction

Human mesenchymal stem cells (hMSC) are isolated from bone marrow samples by using their property to adhere onto a plastic surface [1]. These cells show self-renewal capacity and multipotency [2] characterized by their ability to differentiate to chondrocytes, osteoblasts and adipocytes [3]. The small numbers of these cells in the bone marrow niche [4] makes necessary expanding this stem cell population for therapeutic purposes.

An important characteristic of hMSC is their capability to survive at low densities in the absence of serum [5]. We found that BMP pathway is involved in survival of these cells under these conditions. We also found that, under such serum free conditions, these cells maintain their surface markers profile, their ability to differentiate to mesodermal fates and their ability to proliferate upon serum re-addition to the medium [6]. This rare property of hMSC would allow culturing them in a serum free medium supplemented with growth factors for their expansion or differentiation in the absence of serum. In addition, this culturing approach would allow us studying the effects of specific molecules on cellular processes without interference from serum components.

The molecule chosen to prove this principle was Retinoic acid (RA), which is known by its ability to induce many cellular changes in diverse cell types *in vivo* and *in vitro* [7]–[11]. RA is the oxidized form of Vitamin A that determines anterior/posterior patterning in chordates at early developmental stages [12]. RA acts activating specific gene transcription by binding to heterodimers of the RA receptor (RAR) and the retinoid X receptor (RXR), which then bind to RA response elements (RAREs) in regulatory regions of targets genes [13]. Other two signaling factors used in this study are epidermal growth factor (EGF) and basic fibroblast growth factor (bFGF), which are known to promote cell proliferation and differentiation in diverse kind of cells and tissues via Erk activation [14]–[19]. Here we describe the effects of RA on hMSC biological characteristics under serum free conditions by analyzing the changes at the transcriptome level and verifying such changes at the cell phenotype level. These changes cause downregulation of Erk pathways. Opposite results to those of RA were obtained with EGF and bFGF treatments supporting the role of Erk regulation in the maintenance of hMSC biological properties.

## Results

### Transcriptional effect of RA treatment on hMSC

**Figure 1:**
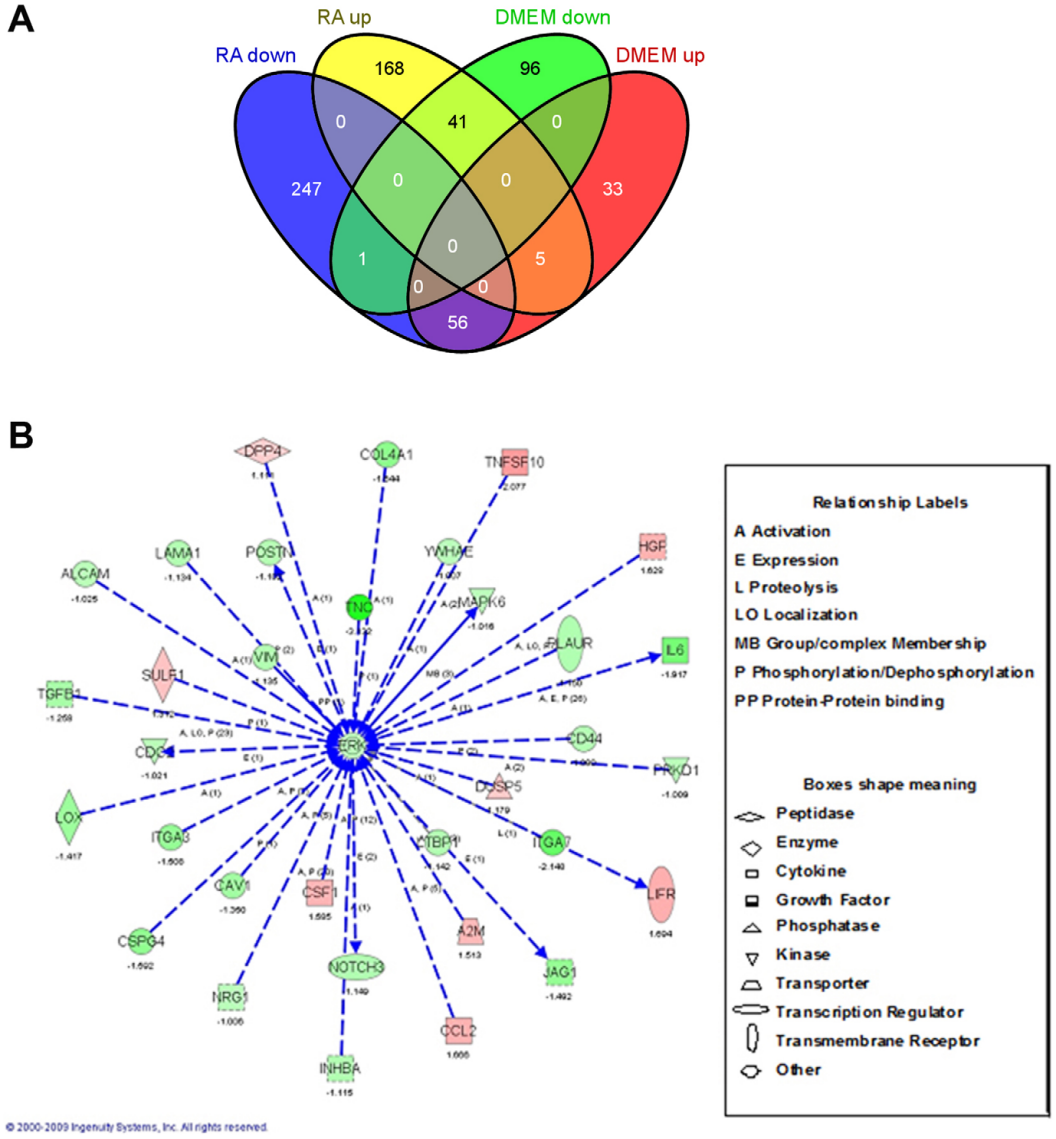
Microarray bioinformatical analysis of the RA effects on hMSC cultured for 5 days without serum. A: Venn diagram showing the number of differentially expressed transcripts in DMEM and RA treated hMSC when compared to 10%FBS treated cells. B: The genes regulated specifically by RA (i.e. blue or yellow in the diagram depicted in A) were clustered by Ingenuity software. This software contains a database with different transcripts arranged in networks according to their known biological interactions. According to the number of transcripts regulated in each of these networks, the program scores them. The best scored network is depicted here. Direct interactions are represented by continuous arrows and direct by dotted ones. Increase in expression is represented by red color and decrease by green. The number under each transcript name is the logarithmic change in comparison to 10%FBS control cells RNA. The type of interaction is indicated by a label and the type of molecule is indicated by the shape of the box, being both detailed at the right of the figure.

Using the ability of these cells to survive and maintain their basic properties under serum free conditions as mentioned above, we investigated the effects of retinoic acid (RA) on hMSC biology under such conditions. Since RA is known by its ability to induce major changes in the cell transcriptome, we performed microarray experiments using the Affymetrix GeneChip Human Gene 1.0 ST oligonucleotide arrays of cDNA produced from hMSC cultured for 5 days in 0.5 µM RA in DMEM or in DMEM with or without 10%FBS. The concentration of RA and the exposure time to such molecule were chosen according to preliminary experiments that showed morphological effects induced by the selected conditions (data not shown). This may suggest that some transcriptional changes may have occurred under such conditions. The microarrays results showed that from the total of transcriptional changes (up or down regulated genes) detected in DMEM or RA compared with 10%FBS, a number of genes were selectively downregulated or up-regulated by RA treatment (168 upregulated, 247 downregulated) indicating RA specific transcriptional regulation in these cells (Figure 1A). To search for the biological meaning of these effects we clustered the RA specifically regulated transcripts (i.e. those changed only by RA treatment and found unchanged in DMEM alone) by using the Ingenuity software (Ingenuity Systems). The resulting network with the best score contained gene transcripts of extracellular proteins involved in cell migration, cell adhesion, and in hMSC differentiation to mesodermal fates, sharing direct or indirect activation of Erk, that were down regulated by RA (Figure 1B). More in detail, adhesion and migration genes down-regulated by RA included ALCAM, CD44, LAMA1, POSTN, ITGA7, ITGA3, TNC, LOX and CSPG4. Differentiation transcripts down-regulated by RA were: POSTN, CSPG4, TGFB1, NRG1, LTBP1, COL4A1, TNC, NOTCH3, JAG1, and INHBA. Some other differentiation related transcripts like HGF, LIFR, and DUSP5 were up-regulated by RA. Moreover, this Erk centralized network also shows some proliferation related transcripts that were down-regulated by RA such as: MAPK6, INHBA, CSPG4, TGB1, CAV1, YWHAE, and CDC2. Altogether, this network analysis suggests the possibility that the RA effects on hMSC that affect diverse cellular properties of hMSC are mediated by ERK dependent pathways.

### RA and EGF+bFGF differently modulate Erk activation

**Figure 2:**
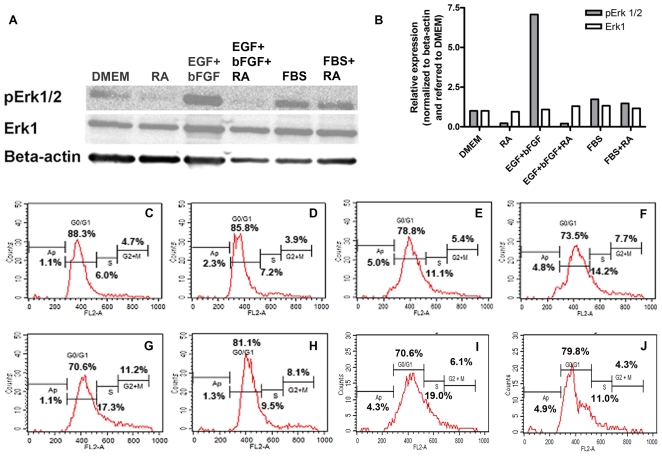
RA and EGF+bFGF effects on Erk phosphorylation and on cell cycle progression. A: Western blot analysis of hMSC that were cultured for 5 days in either DMEM alone (DMEM) or in the presence of 0.5 µM RA (RA) or in the presence of 20 ng/ml EGF+ 5 ng/ml bFGF (EGF+bFGF), with or without the addition of 0.5 µM RA (EGF+bFGF+RA). As control, cells were cultured with 10%FBS (FBS), or with 0.5 µM RA in 10%FBS (FBS+RA). Whole-cell protein extracts from these differently treated cells were fractionated on a denaturating 12% polyacrylamide gel, transferred to nitrocellulose and detected with anti phosphorylated Erk antibody (pErk1/2). The membrane was stripped twice, one for detection with anti total Erk antibody (Erk1) and the second for β-actin antibody detection used as loading control. B: Densitometry analysis of A. The bars represent relative expression normalized to β-actin expression and referred to this ratio in DMEM. C–J: Cell cycle progression by FACS of hMSC that were cultured for 5 days with DMEM (C), 0.5 µM RA in DMEM (D), 20 ng/ml EGF (E), 5 ng/ml bFGF (F), 5 ng/ml bFGF +20 ng/ml EGF (G), or 5 ng/ml bFGF +20 ng/ml EGF +0.5 µM RA (H). In addition, hMSC were cultured for 2 days in DMEM (I) or in the presence of 0.5 µM RA (J) before replacement of the medium with 20 ng/ml EGF +5 ng/ml bFGF in DMEM for further 2 days. At the end of the experiment the cells were harvested by trypsinization, permeabilized and stained with propidium iodide to measure the DNA content by FACS.

To challenge this hypothesis we started by confirming the effects of RA on the Erk pathway as suggested by the microarray analysis. We tested Erk activation in the presence and absence of RA and under known Erk activating conditions on hMSC. To this end we made protein lysates from cells cultured for 5 days in DMEM, in 10% FBS or in 20 ng/ml EGF+ 5 ng/ml bFGF with or without 0.5 µM RA. Western blot analysis was performed using antibodies against human phosphorylated Erk1/2 and against total Erk1 protein. β-actin was used for normalization in densitometry analysis. The results from these experiments are shown in Figure 2A–B. The presence of EGF+bFGF increases Erk1/2 phosphorylation while RA blocks this inducing effect whereas the expression of total Erk1 remains unaffected. Under serum presence Erk1/2 phosphorylation is moderately induced (compared to EGF+bFGF effect) and the RA inhibitory effect, which is clearly visualized in DMEM with or without EGF+bFGF, is undetectable. This suggests that Erk activation under serum is modulated probably by a mix of positive and negative factors while EGF+bFGF or RA in DMEM elicit Erk activation or inhibition in a direct manner respectively. These results demonstrate that hMSC cultured under serum free conditions provide a platform to specifically characterize a signaling pathway in these cells.

### RA and EGF+bFGF have opposite effects on hMSC proliferation

Having established the effects of RA and EGF+bFGF on Erk activation we wanted to evaluate their effects on hMSC proliferation. To this end, we analyzed the cell cycle by FACS analysis of hMSC cultured for 5 days in DMEM alone or in combination with 20 ng/ml EGF+ 5 ng/ml bFGF and with or without 0.5 µM RA. Figure 2C–H shows that RA decreases cell cycle progression by increasing the proportion of cells in G0/G1 while in contrast EGF and bFGF combination promotes cell cycle progression synergistically. These opposite effects between RA and EGF+bFGF are consistent with their opposite effects on Erk activation as shown above. At the next step we wanted to know whether this antiproliferative RA effect is reversible. To this end we performed similar FACS analysis on hMSC cultured for two days either in DMEM or in 0.5 µM RA in DMEM followed by further two days culture in 20 ng/ml EGF+ 5 ng/ml bFGF in DMEM after thorough washes. Figure 2I–J indicates that the blocking effect of RA on hMSC proliferation is maintained even if RA is removed before EGF+bFGF treatment. This suggests that the transcriptional changes of proliferation genes induced by RA on hMSC as described in Figure 1 may have irreversible impact on the proliferative capacity of these stem cells.

### Effects of RA, EGF+bFGF and laminin coating on hMSC adhesion and cell migration

**Figure 3:**
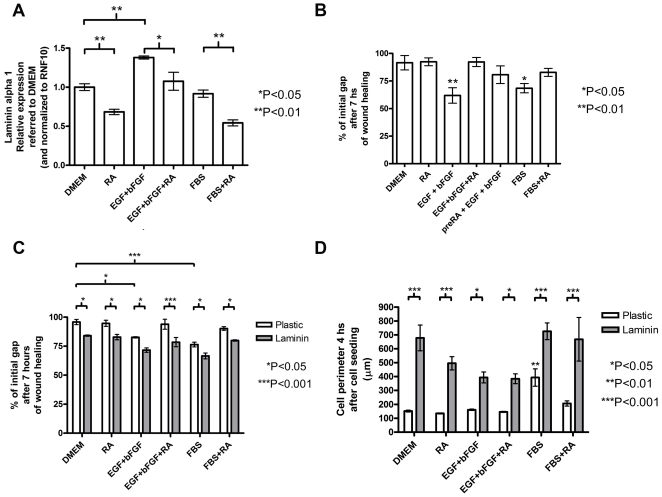
RA and EGF+bFGF treatments affect laminin expression which modulates cell migration and adhesion of hMSC. A: QRT-PCR analysis of laminin alpha 1 expression in hMSC that were treated for 5 days with either DMEM, 0.5 µM RA in DMEM (RA), 20 ng/ml EGF+ 5 ng/ml bFGF (EGF+bFGF), 20 ng/ml EGF +5 ng/ml bFGF +0.5 µM RA (EGF+bFGF+RA), 10%FBS (FBS), or 0.5 µM RA in 10%FBS (FBS+RA). The error bars represent relative expression normalized to RNF10 expression and referred to the relative expression on DMEM as mean ± s.e.m. B: Wound healing experiments were performed on hMSC that were previously treated for 5 days as in A and also treated with 0.5 µM RA followed by EGF+bFGF at the time of the experiment (preRA+EGF+bFGF). The error bars represent % of the initial gap after 7 hours as mean ± s.e.m. C: Similar wound healing experiments as shown in B but hMSC were previously seeded over either laminin coated (20 µg/well) (laminin) or uncoated plates (plastic) and treated as in A. The error bars represent % of the initial gap after 7 hours as mean ± s.e.m. D: Cell spreading assay of hMSC that were treated for 5 days similarly as in A (see Materials and Methods). The error bars represent cell perimeter as mean ± s.e.m. All the treatments in A–D were performed in triplicates in two independent experiments with different hMSC donors and the significance of the results was assessed using one way ANOVA and Tukey's multiple comparison test (for A and B) or with two way ANOVA followed by Bonferroni post test (for C and D). *P<0.05; **P<0.01; ***P<0.001.

Following the microarray results, the Laminin alpha 1 (LAMA1) transcript which is down-regulated by RA, and connected to the ERK pathway (see Figure 1B), was chosen for validating the RA effects given its known relation with cell migration and adhesion [20]. In these experiments we validated the effects of RA on LAMA1 transcription levels by QRT-PCR analysis of cDNA samples prepared from RNA obtained from hMSC treated for 5 days in the presence or absence of RA. In parallel, following the EGF+bFGF effects on Erk activation and cell proliferation in the presence or absence of 0.5 µM RA (see Figure 2) we performed QRT-PCR analysis of LAMA1 expression in hMSC treated for 5 days with 20 ng/ml EGF+ 5 ng/ml bFGF combination or with 10% FBS, both in the presence and in the absence of RA. Figure 3A shows the results from these experiments. These results confirmed the microarray results reflecting that RA causes a significant decrease in LAMA1 expression. In a similar way as shown for Erk activation (Figure 2) we observed that EGF+bFGF treated cells increased LAMA1 expression while together with RA this effect was blocked. Similarly as shown above for Erk activation, 10%FBS did not affect significantly LAMA1 expression compared to DMEM alone but in the presence of RA the expression of LAMA1 decreased significantly. To study whether these treatments affect cell migration in hMSC we performed wound healing assays. To this end we cultured hMSC for 5 days in DMEM alone or with the addition of 20 ng/ml EGF+ 5 ng/ml bFGF or 10%FBS in the presence or absence of 0.5 µM RA. After this period a wound on the different treated cultures was made mechanically and the closure of the gap by the migrating cells was followed for seven hours by photographing the area every hour under an inverted microscope. Figure 3B shows the analysis from this experiment at the end time point (7 hs) indicating that whereas EGF+bFGF or FBS promote cell migration, cells in RA or DMEM are not able to migrate significantly. RA dramatically reduces the positive effect of EGF+bFGF and of FBS on cell migration. Moreover, even 5 days pre-treatment with RA before EGF+bFGF (preRA+EGF+bFGF) at the time of the wound healing experiment is able to reduce the migration of hMSC. Altogether these experiments demonstrate that RA and EGF+bFGF have an opposite effect on LAMA1 expression that could be reflected by their respective effects on cell migration. To investigate whether LAMA1 is required for hMSC migration we performed a wound healing experiment as described above but this time hMSC were cultured on either a laminin coated (20µg/well) or uncoated plate for 5 days under the same treatments described above (see Figure 3A). The results from these experiments are shown in Figure 3C demonstrating an increase in cell migration which closes the gap faster when the cells are cultured in a laminin-coated plate for all the treatments, suggesting that laminin is required for hMSC migration. Moreover, laminin can rescue the inhibitory effect of RA on cell migration strongly suggesting that laminin elicits intracellular signaling pathways that drive cell migration overriding the negative RA effect. Altogether, these results strongly supports that the RA effect on laminin downregulation directly affects hMSC migration capacity.

As mentioned before, laminin is also involved in cell adhesion [20]. For this reason, we were interested to assess the laminin role in hMSC adhesion and whether this process is affected by RA. To this end, we carried out spreading assays after culturing hMSC in different media treatments for 5 days in a similar way as stated above (see Figure 3A). We harvested each of the treated cells using trypsin, and cultured them for 30 minutes in suspension with the same medium to allow cell matrix protein recovery. Then, we plated the cells in suspension onto an uncoated plate or onto a laminin-coated one (20 µg/well), and then the cells were photographed under an inverted microscope in order to analyze their perimeter after 4 hours with the aid of image analysis software. The results from these experiments (shown in Figure 3D) indicate that serum, but not EGF+bFGF, is required for hMSC spreading, whereas RA decreases this cell adhesion process. In addition, laminin is enough by itself to induce hMSC spreading enhancing cell adhesion, even in the absence of serum and in the presence of RA. Again, here we demonstrate that the RA effect on Laminin expression directly affects hMSC adhesion properties.

### Effects of RA, EGF+bFGF and Periostin on hMSC osteogenesis

**Figure 4:**
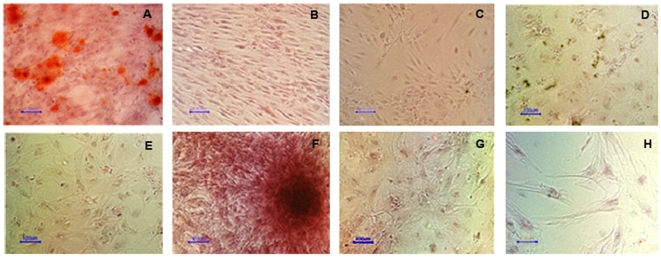
hMSC osteogenesis is inhibited by RA and enhanced by EGF+bFGF. hMSC were cultured for 21 days in osteoblasts differentiation medium without serum supplemented with 10% FBS (A), 10% FBS +0.5 µM RA (C), nothing (D), 0.5 µM RA (E), 20 ng/ml EGF +5 ng/ml bFGF (F), 20 ng/ml EGF +5 ng/ml bFGF +0.5 µM RA (G), or were cultured in DMEM supplemented with 10% FBS (B) or with 20 ng/ml EGF +5 ng/ml bFGF (H) (negative controls). To visualize differentiation osteoblasts were stained with alizarin red after fixation in 70% ethanol. Size bars = 100 µm.

So far we have shown that RA affects all the hMSC biological characteristics we have tested and that the EGF+bFGF combination promotes most of them. Therefore, we wanted to investigate the possibility that another characteristic of hMSC like cell differentiation could be affected by these factors too. To this end, hMSC were cultured in osteoblasts differentiation medium for 21 days in the presence or absence of 10% FBS or supplemented with 20 ng/ml EGF +5 ng/ml bFGF instead FBS, with or without 0.5 µM RA. DMEM supplemented with 20 ng/ml EGF +5 ng/ml bFGF served as a control to test if these factors alone promote osteogenesis. To visualize osteoblast differentiation the cultures were stained with alizarin red at the end of the experiments. The results from these experiments are shown in Figure 4 and show that RA blocks osteogenesis induced either by serum or by EGF+bFGF combination. Interestingly, here we show for the first time that EGF+bFGF combination replaces the use of serum in the osteogenesis protocol when the other factors of the osteogenic medium (dexamethasone, vitamin C and β-glycerol phosphate) are present.

**Figure 5:**
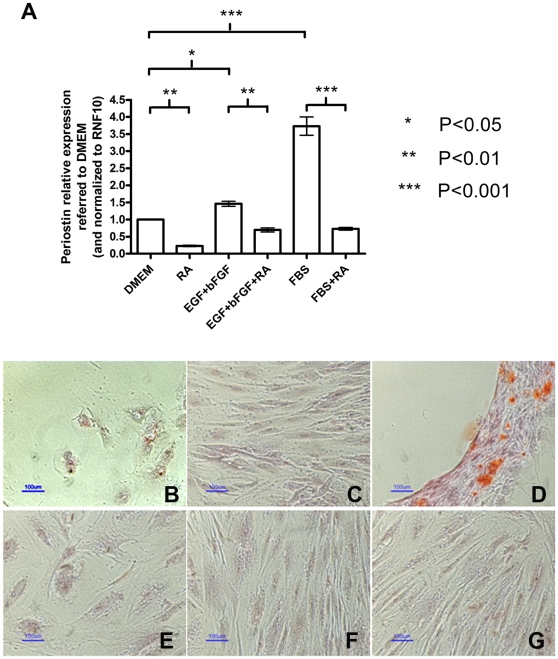
Periostin can rescue the effect of RA on hMSC osteogenesis. A: hMSC were treated for 5 days with either DMEM, 0.5 µM RA in DMEM (RA), 20 ng/ml EGF+ 5 ng/ml bFGF (EGF+FGF), 20 ng/ml EGF +5 ng/ml bFGF +0.5 µM RA (EGF+FGF+RA), 10%FBS (FBS), or 0.5 µM RA in 10%FBS (FBS+RA). The RNAs were purified and the cDNAs were synthesized using reverse transcriptase. Periostin transcript was quantified in each sample. The error bars represent relative expression normalized to RNF10 expression and referred to the relative expression on DMEM as mean ± s.e.m. All the treatments were performed in triplicates in two independent experiments with cDNA from different donors and the significance of the results was assessed using one way ANOVA and Tukey's multiple comparison test. B–G: hMSC were cultured in osteoblasts differentiation medium supplemented with 0.5 µM RA (B–D), or in 10%FBS +0.5 µM RA (negative control) (E–G), for 21 days over uncoated plastic (B and E), 2.5 µg laminin/well coated plastic (C and F), or 2.5 µg periostin/well coated plastic (D and G). Osteoblasts were stained with alizarin red. Size bars = 100 µm.

Microarray analysis results of hMSC (see Figure 1) suggested Periostin (POSTN) as a possible downregulated target of RA. Periostin is believed to be involved in the mineralization of the extracellular matrix that takes place during osteogenesis [21]. Therefore, it was important to validate this result, and at the same time, since POSTN is known to be upregulated by Erk, to verify whether EGF+bFGF and even serum are able to increase its expression. To this end, we performed QRT-PCR analyses with cDNA obtained from RNA purified from cells obtained from different donors cultured 5 days in either DMEM, 0.5 µM RA, 20 ng/ml EGF+ 5 ng/ml bFGF, EGF+bFGF+RA, FBS, or FBS+RA. The results from these experiments are shown in Figure 5A and indicate that indeed periostin expression is down-regulated by RA and up-regulated by EGF+bFGF or by serum. Moreover, RA brings down its expression even in cells treated with EGF+bFGF or serum. These results confirm the microarray results for the RA effects on periostin expression and most importantly they suggest that periostin may be playing a role in hMSC osteoblast differentiation potential since its expression can be positively regulated either by EGF+bFGF or by serum.

Following the negative effect observed on the osteogenetic potential of these cells by RA and the effect on the expression of periostin induced by this molecule, it was interesting to assess whether periostin coating could alleviate the negative effect of RA on osteogenesis of hMSC. For this purpose, we performed osteoblast differentiation protocol as described above in the presence of 0.5 µM RA but on uncoated plates or coated either with 2.5 µg periostin/well or 2.5 µg laminin/well. Laminin coating was used as matrix specificity control for differentiation. The results from this experiment are shown on Figure 5B-G and indicate that indeed periostin is able to rescue hMSC from the negative effect of RA on their osteogenetic potential whereas laminin has no effect on it. This demonstrates the need and specificity of periostin for osteogenesis and that this extracellular driven signaling pathway overrides RA's inhibition of differentiation.

### Effects of RA and EGF+bFGF on hMSC adipogenesis

**Figure 6:**
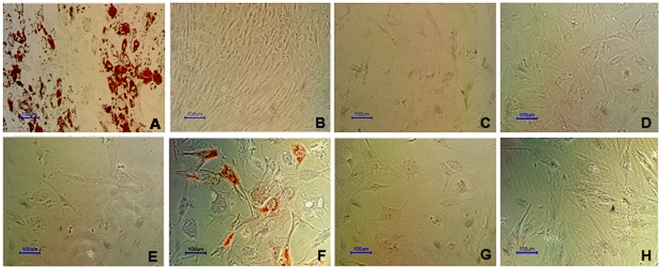
hMSC adipogenesis is inhibited by RA and enhanced by EGF+bFGF. hMSC were cultured for 21 days in either adipocytes maintenance medium (days 7–8 and 15–16) or adipocytes induction medium (the rest of the days) (see Materials and Methods for seeing the media composition), without serum, supplemented with 10% FBS (A), 10% FBS +0.5 µM RA (C), nothing (D), 0.5 µM RA (E), 20 ng/ml EGF +5 ng/ml bFGF (F), 20 ng/ml EGF +5 ng/ml bFGF +0.5 µM RA (G). In addition, hMSC were cultured in DMEM supplemented with 10% FBS (B) or with 20 ng/ml EGF +5 ng/ml bFGF (H) (negative controls). To visualize differentiation, adipocytes were stained with Oil red O after fixation with 4% paraformaldehyde. Size bars = 100 µm.

Having observed that RA inhibits differentiation of hMSC to osteoblasts and that EGF+bFGF enhance such process, it was important to determine if another cell fate potential of hMSC is affected by these molecules. To this end we performed similar treatments as shown in Figure 5 using suitable culture media for adipogenesis. In these experiments we observed, in a similar way as for osteogenesis, that RA inhibits and EGF+bFGF enhance the adipogenesis process (see Figure 6).

## Discussion

Here we exploit the fact that culturing hMSC under serum free conditions allows studying the specific effects on these cells of factor/s added to the medium. This approach gave us the possibility to investigate the effects of RA on hMSC biological properties. We found that RA treatment inhibits the transcription of specific genes that converge in Erk inactivation. This effect is visualized by a decrease in typical properties of hMSC such as cell migration, adhesion, proliferation and loss of differentiation potential to both osteoblasts and adipocytes. In contrast to these results we found that EGF and bFGF combination strengthens the mesenchymal properties of these cells promoting cell migration, proliferation and differentiation of hMSC to such fates. Microarray analyses suggested that RA down regulated genes that are biologically related to Erk regulatory pathways (Figure 1). These results were confirmed by Western blot analysis showing that after 5 days exposure to RA the phosphorylation of Erk is decreased. In contrast, EGF+bFGF combination up-regulates Erk phosphorylation but RA is capable to decrease it even in the presence of EGF+bFGF (Figure 2A–B). Altogether, these findings strongly indicate that Erk is regulated by the extracellular factors RA and EGF+bFGF that elicit opposite phenotypic effects on the hMSC biological characteristics mentioned above. To the best of our knowledge this is the first evidence showing the importance of these signaling factors in hMSC biology and the implication of the Erk pathways in these adult stem cell characteristics.

Although we have not deciphered yet the mechanism responsible for the RA effect on Erk phosphorylation, results from the microarray experiment supports that RA directly affects this signaling pathway since several known activators of Erk such as COL4A1 [22], CD44 [23], CAV1 [22], CSPG4 [24], ITGA3 [25], TGFB1 [26], ALCAM [27], VIM [28], LAMA1 [22] and TNC [29] were also down-regulated by RA (Figure 1).

Interestingly, RA affects negatively the proliferation capacity of hMSC which is promoted by either EGF or bFGF or by a combination of both factors (Figure 2C–H). Pre-incubation with RA decreases the ability of hMSC to proliferate even in the presence of EGF+bFGF (Figure 2I–J). This may indicate that RA causes irreversible changes in the cell transcriptome that influence also the proliferative capacity even after its removal. Our findings are in accordance with previous works showing enhancement of hMSC proliferation by EGF and bFGF [30]. However, this is the first time that the opposite effects of RA and EGF+bFGF are shown at the proliferation level in parallel to the Erk activation in a serum free context.

We showed that RA also decreases hMSC migration which is increased by EGF+bFGF. This process is also promoted by serum and by laminin alone (Figures 3B–C). These findings are in accordance with the effect of each of these treatments in the expression of LAMA1 (laminin 1), as shown by QRT-PCR analysis (Figure 3A) and with our microarray analysis (Figure 1). Given that laminin rescues the RA effect, this means that possibly laminin activates some integrin mediated intracellular signals that may transduce in Erk activation. This is in accordance with a recent work showing a positive effect of Laminin1 on Erk activation and on cell spreading in hMSC from bone marrow under serum free conditions [31].

Pretreatment of hMSC with RA also impairs cell migration even before the addition of EGF+bFGF (Figure 3B). The fact that RA induces effects that are sustained after its removal from the medium supports the view that RA causes transcriptional changes in the cell that decrease also the potential of hMSC to migrate. Another typical characteristic of hMSC that has been affected by RA treatment is cell adhesion. We showed that laminin alone restores the ability of these cells to adhere (Figure 3D). Interestingly here, EGF+bFGF did not have an apparent effect on cell spreading. This in fact strengthens the central role of laminin to drive cell adhesion in these stem cells which may activate an independent signal transduction pathway to that of EGF+bFGF.

We found that RA inhibits osteogenesis. In addition, when we cultured the cells with differentiation medium but without serum, the differentiation did not take place. However, when EGF+bFGF were added to this serum free osteogenic medium it enhanced notably this differentiation (Figure 4). Our results are in accordance with other authors [32], [33] claiming that Erk activation enhance osteogenesis in hMSC. However, this is the first report of a totally serum free osteogenesis protocol that could be developed for therapeutic purposes in the future.

We found that periostin expression is decreased by RA according to our microarrays (Figure 1). QRT-PCR confirmed this and also demonstrated that EGF+bFGF increase periostin expression (Figure 5A). This suggests that periostin may be playing a role in hMSC osteogenesis since its expression can be positively regulated either by EGF+bFGF or by serum (which promote osteogenesis) and down-regulated by RA (which inhibits this process). Following this, we determined that periostin is able to rescue the negative effect of RA on osteogenesis when supplied exogenously (Figure 5B–G). This result demonstrates the need and specificity of periostin for osteoblast differentiation promotion and that this extracellular driven signaling pathway overcomes RA's inhibition of differentiation. It is important to note that whereas periostin is able to rescue the RA effect on osteogenesis, laminin is able to rescue the effect of RA on adhesion but not on osteogenesis. This is in accordance with the role of each of these proteins and suggests that laminin can be important to maintain the stem cell status prior to differentiation whereas periostin seems to have a role at the end of the differentiation, specifically in the mineralization process [21].

Studies have shown that mitogen-activated protein kinase (MAPK) [34], [35] and phosphatidylinositol 3-kinase (PI-3K) [36] activity are critical switches for osteogenic differentiation. In addition, the study from Seib *et al*. further demonstrates that spontaneous osteogenesis in human MSCs is regulated through these signaling pathways, which can be induced by endogenous BMPs [37]. These works coincide with our Western blots from lysates of hMSC treated with EGF+bFGF (Figures 2A–B) which show that this factor's combination promotes Erk phosphorylation, but also up-regulates PI-3K as judged by Akt phosphorylation (data not shown).

The results showing RA inhibition of adipogenesis and EGF+bFGF enhancement of this process (see Figure 6) further support the opposite effects of these molecules on the hMSC biology. Interestingly, osteogenesis and adipogenesis are equally affected by these molecules.

It is possible that the loss of hMSC properties like cell survival, cell adhesion, cell migration, and differentiation to mesodermal fates induced by RA treatment are also accompanied by a change in the cell status of hMSC. To verify this possibility we checked in our microarray analysis the effect of RA in hMSC identity. We found that 11.5% of the upregulated transcripts are related to ectodermal lineage while 10.1% of the downregulated transcripts are from mesodermal origin. This fact certainly suggests that RA is inducing these cells to a more ectodermal state of differentiation. This suggestion is in accordance with the known ability of RA to induce neuroectodermal differentiation [38].

The present study opens the possibility to use bone marrow hMSC in serum free conditions for therapeutic purposes. Since we show the principle that the basic properties of these cells do not change with serum deprivation, it might be possible theoretically to expand them (using for example EGF+bFGF) and differentiate them to either adipocytes or osteoblasts without serum. In addition, we show the principle that these cells may serve as a platform for studying specifically the effects of different molecules on their cell biology such as RA and/or EGF+bFGF. Therefore, this study is important for the fields of regenerative medicine and cell biology.

## Materials and Methods

### Ethics statement

Samples were obtained from the iliac crest's bone marrow of healthy donors (20 to 65 years old) who had signed for written consent according to the guidelines of the ethics committee of the Laniado Hospital (supervised by the Israeli Health Ministry) which specifically approved this study.

### hMSC isolation and culture

The bone marrow aspirates were diluted 1:2 with Hanks' Balance Salt Solution (Biological Industries, Israel). The mononuclear cell layer was isolated by density gradient using UNI-SEP maxi tubes (Novamed, Israel) centrifuged at 1000 g for 20 minutes, and resuspended in 6 volumes of medium (referred from here onwards as FBS). FBS was composed of DMEM (Gibco, Invitrogen, NY, USA) supplemented with 1 mM MEM Sodium Pyruvate (Gibco, Invitrogen, NY, USA), 1% Penicillin-streptomycin-nystatin solution (Biological Industries, Israel), and 10% FBS (Hyclone, USA). The cells were cultured in polystyrene plastic 75 cm^2^ tissue culture flasks (Corning, NY, USA) at 37°C with 5% CO_2_. hMSC were isolated by their characteristic plastic adherence while other cells were washed through successive medium replacement as previously described [39]. Cell passages were made when cells reached 80–90% confluency (∼1.2×10^6^ cells/flask). hMSC from passages 2 to 7 were used for the different experiments.

### Differentiation to osteoblasts

hMSC were plated (60000 cells/well) in 24 wells plates and grown to confluence. Then the cells were cultured during 14–17 days in a medium (changed every 2–3 days) composed of DMEM (Gibco, Invitrogen, NY, USA) supplemented with 1 mM MEM Sodium Pyruvate (Gibco, Invitrogen, NY, USA), 1% Penicillin-streptomycin-nystatin solution (Biological Industries, Israel), 0.1 µM dexamethasone (Sigma, MO, USA), 0.2 mM ascorbic acid 2-phosphate (Sigma, MO, USA), and 10 mM glycerol 2-phosphate (Sigma, MO, USA). The medium was employed with or without 10% FBS (Hyclone, USA), with or without 0.5 µM RA, and with or without a combination of 20 ng/ml EGF +5 ng/ml bFGF. The obtained osteoblasts were fixed in 70% ethanol and stained with Alizarin Red (Sigma, MO, USA).

### Differentiation to adipocytes

hMSC were cultured during 21 days in 24 well plates at 60000 cells/well using the following medium change schedule since 100% confluency was reached: days 1, 3, 5: induction medium; day 7: maintenance medium; days 9, 11, 13: induction medium; day 15: maintenance medium; days 17, 19, 21: induction medium. Maintenance medium was composed of DMEM (Gibco, Invitrogen, NY, USA) supplemented with 1 mM MEM Sodium Pyruvate (Gibco, Invitrogen, NY, USA), 1% Penicillin-streptomycin-nystatin solution (Biological Industries, Israel), 10% heat inactivated FBS (Hyclone, USA), and 10 µg/ml insulin (Sigma, MO, USA). Induction medium was composed of maintenance medium supplemented with 1 µM dexamethasone (Sigma, MO, USA), 0.5 mM IBMX (Sigma, MO, USA), and 100 µM indomethacine (Sigma, MO, USA). The resulting adipocytes were fixed in 4% paraformaldehyde and stained with Oil Red O solution (Sigma, MO, USA).

### FACS for cell cycle analysis

Cultured hMSC were collected by trypsinization. The cells were re-suspended in PBS and transferred to a FACS tube, centrifuged and re-suspended again in PBS. Then 0.1% triton X-100 and 20 µg/ml PI were added to stain the cell nuclei. The cell cycle pattern of the PI stained material was analyzed by a FACSORT™ (Becton Dickinson) flow cytometer using an argon laser at 488 nm.

### Microarray analysis

All experiments were performed using Affymetrix GeneChip Human Gene 1.0 ST oligonucleotide arrays. 100–600 ng of total RNA from each sample, purified by TRIzol reagent (Invitrogen, USA), was used to prepare biotinylated target DNA that was processed following manufacturer's recommendation, using an Affymetrix GeneChip Instrument System. The quality of starting RNA was confirmed using agarose gel electrophoresis. Ingenuity IPA software was used to build protein interaction networks between the differentially regulated genes.

### Quantitative Real-Time PCR

QRT-PCR assays were carried out in triplicates on a Rotor-Gene 6000 real-time rotator analyzer (Corbett Research) using the Absolute QPCR SYBR green (Thermo Scientific) according to manufacturer's instructions. The different pairs of specific primers (Table 1) were added at a final concentration of 200 nM in a 10 µl reaction volume mix containing 1.5 µl cDNA. Thermo-cycling conditions were: 95°C for 15 minutes followed by 45 cycles at 95°C for 10 seconds, 56°C for 15 seconds and 72°C for 20 seconds. At the end of the process a melt analysis step ranging from 72°C to 95°C was added to assess the specificity of the reaction. The threshold cycle number (Ct) was determined for all PCR reactions by automatically adjusting the threshold within the logarithmic curve, using Rotor-Gene 6000 series software (Corbett Research). A non-template control was included for each gene. A concentration vs. Ct curve was build for each gene using known dilutions from a cDNA mix of all the samples. The relative concentration of each gene in each sample, calculated by interpolating its Ct in its respective curve, was normalized to the relative concentration of RNF10 in the same sample.

**Table 1:** Primers used for QRT-PCR in this study.

Gene	Forward 5′ to 3′	Reverse 5′ to 3′
LAMA1	CAGGACCCATTACCCTTTTG	GCCCTGCTTGGTTTCTTTATT
Periostin	CCCTATAAACGCCAACAATCA	TCTGTTGAAGGGCACAGACA
RNF10	AAGGGAGGTCACTGGTGTTG	CTTCATCGAAGGCAGACAGA

### Western blot

Cell lysates were obtained with RIPA buffer (Sigma) in the presence of the “Complete” protease inhibitor (Roche). The lysates were sonicated, spun down at 9000 g and the protein concentration was calculated in each supernatant by BCA protein assay kit (Pierce, IL, USA). A 12% SDS-PAGE was loaded with 11.5 µg protein from each sample after boiling for 10 minutes, and all the loaded proteins were electrophoresed and then transferred onto a nitrocellulose membrane. After 1 hr blocking at room temperature in blocking buffer [5% non fat milk in Tris-buffered saline/1% tween 20 (TBS/T)] and after washing three times for 5 minutes each in TBS/T, the membranes were incubated overnight at 4°C with the primary antibody (Table 2) diluted in 5% Bovine serum albumin in TBS/T. Following three washes with TBS/T, secondary antibody (Table 2) diluted 1∶4000 in blocking buffer was added for 1 hr at room temperature and washed three times with TBS/T. The detection was performed by ECL reaction. All the signals were quantified by normalizing to the β-actin signal.

**Table 2:** Antibodies used for Western blot in this study.

Antibody	Raised in	Dilution
anti Phospho-p44/42 MAPK (Erk1/2) (Thr202/Tyr204) (Cell signaling technologies, USA)	Rabbit	1∶1000
anti Erk1 (Zymed, CA, USA)	Mouse	1∶500
anti human β-actin (MP Biomedicals, OH, USA)	Mouse	1∶2000
anti-rabbit IgG (H + L) HRP conjugate (Jackson Immunoresearch Laboratories, PA, USA)	Donkey	1∶4000
anti-mouse IgG (H + L) HRP conjugate (Jackson Immunoresearch Laboratories, PA, USA)	Donkey	1∶4000

### Wound healing assay

Wound healing experiments were performed on confluent cultures of hMSC treated for 5 days under different conditons. Following the treatments a scratch was mechanically made to produce a gap on the cell layer. The cells were photographed at the gap area to visualize and measure changes in the gap width due to cell migration, at the beginning and 7 hours after the wound was made. The percent of the initial gap after 7 hours was determined by the ratio between the average widths measured at several points along the gap in the images from the two time points.

### Cell spreading assay

hMSC were treated for 5 days with different conditions. Then, the cells were collected by trypsinization and were incubated in suspension for 30 minutes at 37°C with the same media to allow them to regenerate adhesion molecules. Then the cells were plated back on 24 well-plates either uncoated or coated with 20 µg laminin per well. Following 4 hours of incubation, the cells were photographed. Cell perimeter was measured for each cell in the population using Slidebook™ (Intelligent imaging, Inc.) software as parameter for assessing cell adhesion under the different culture conditions tested.
